# Efficacy of Topical Treatment with (−)-Epigallocatechin Gallate, A Green Tea Catechin, in Mice with Cutaneous Leishmaniasis

**DOI:** 10.3390/molecules25071741

**Published:** 2020-04-10

**Authors:** Andrea M. Sosa, Agustín Moya Álvarez, Estefanía Bracamonte, Masataka Korenaga, Jorge D. Marco, Paola A. Barroso

**Affiliations:** 1 Instituto de Patología Experimental, Facultad de Ciencias de la Salud, Universidad Nacional de Salta-CONICET, Salta 4400, Argentina; ams_868@yahoo.com.ar (A.M.S.); elagus177@gmail.com (A.M.Á.); tefybracamonte@gmail.com (E.B.); diegomarcoar@gmail.com (J.D.M.); 2 Department of Parasitology, Kochi Medical School, Kochi University, Okocho Kohasu, Nankoku, Kochi Prefecture 783-8505, Japan; korenaga@kochi-u.ac.jp; 3 Faculty of Health Sciences, Kochi Gakuen University, Asahi-Tenjincho, Kochi, Kochi Prefecture 780-0955, Japan

**Keywords:** green tea catechins, *Leishmania (L.) amazonensis*, cutaneous leishmaniasis, (−)-epigallocatechin gallate

## Abstract

The treatment of leishmaniasis includes pentavalent antimony drugs but, because of the side effects, toxicity and cases of treatment failure or resistance, the search of new antileishmanial compounds are necessary. The aims of this study were to evaluate and compare the in vitro antileishmanial activity of four green tea catechins, and to assess the efficacy of topical (−)-epigallocatechin gallate in a cutaneous leishmaniasis model. The antileishmanial activity of green tea catechins was evaluated against intracellular amastigotes, and cytotoxicity was performed with human monocytic cell line. BALB/c mice were infected in the ear dermis with *Leishmania (Leishmania) amazonensis* and treated with topical 15% (−)-epigallocatechin gallate, intraperitoneal Glucantime, and control group. The efficacy of treatments was evaluated by quantifying the parasite burden and by measuring the lesions size. (−)-Epigallocatechin gallate and (−)-epigallocatechin were the most active compounds with IC_50_ values <59.6 µg/mL and with a selectivity index >1. Topical treatment with (−)-epigallocatechin gallate decreased significantly both lesion size and parasite burden (80.4% inhibition) compared to control group (p < 0.05), and moreover (−)-epigallocatechin gallate showed a similar efficacy to Glucantime (85.1% inhibition), the reference drug for leishmaniasis treatment.

## 1. Introduction

Cutaneous leishmaniasis (CL) is a widespread tropical infection caused by a protozoa parasite of *Leishmania* genus which is transmitted to humans by the bite of an infected sand fly [1]. In the New World, CL is produced by *Leishmania (Leishmania) mexicana*, *L. (L.) amazonensis*, *L. (Viannia) braziliensis*, *L. (V.) panamensis*, *L. (V.) peruviana*, and *L. (V.) guyanensis.* In general, CL is characterized by an ulcerative lesion (single or multiple), and depending of *Leishmania* species, the disease can progress to more severe manifestations such us mucocutaneous, diffuse leishmaniasis, disseminated leishmaniasis, and leishmaniasis recidivans [1]. The antimonials, sodium stibogluconate (Pentostam) or meglumine antimoniate (Glucantime), are the first choice for the treatment of all forms of leishmaniasis, but because of their toxicity, high cost and prolonged treatment regimens, are not satisfactory as an ideal drug. In addition, treatment failure was reported in a clinical trial where meglumine antimoniate showed a low efficacy against *Leishmania Viannia* species [2]. Therefore, there is a need for new antileishmanial drugs. 

The green tea from the leaf of *Camellia sinensis* (Theaceae) is one of the most popular beverages worldwide, and for many years the health benefits of its polyphenol E (PE) fraction have been studied. The PE is constituted by (+)-catechin (C), (−)-epicatechin (EC), (−)-gallocatechin (GC), (−)-epigallocatechin (EGC), (−)-gallocatechin gallate (GCG), (−)-catechin gallate (CG) and (−)-epigallocatechin gallate (EGCG), which is the major one among of these components. The PE fraction has antioxidant activity, anti-inflammatory, antitumoral and antimicrobial effects [3,4,5]. In addition, EGCG administered by intraperitoneal injection showed anti-*Trypanosoma cruzi* activity decreasing significantly the parasite burden and increasing the mice survival [6]. These were consistent with the inhibition of *T. cruzi* amastigote replication in infected Vero cells by EGCG and GCG [7]. Then, our group studied the antileishmanial effect of seven catechins and the whole PE fraction from *C. sinensis* against two species, *L. (L.) amazonensis* and *L. (V.) braziliensis* [8]. EGCG, GCG, EGC, GC and GCG inhibited the growth of promastigotes of both *Leishmania* species, and C and EC were the less active compounds [8]. Moreover, the PE fraction showed activity against intracellular amastigotes of *L. (L.) amazonensis* [8]. Recently, it was reported that EGCG oral treatment showed high efficacy inhibiting the parasite burden of *L. (V.) braziliensis* (92.1%) and *L. (L.) amazonensis* in BALB/c mice [9,10]. Moreover, the antileishmanial activity of EGCG is related to the generation of oxygen reactive species (ROS) that alter the mitochondrial function and kill the parasite [11]. Another mechanism of action of EGCG is related to its capacity to inhibit leishmania arginase, increasing the nitric oxide toxic agent for the parasite [12]. 

**Figure 1:**
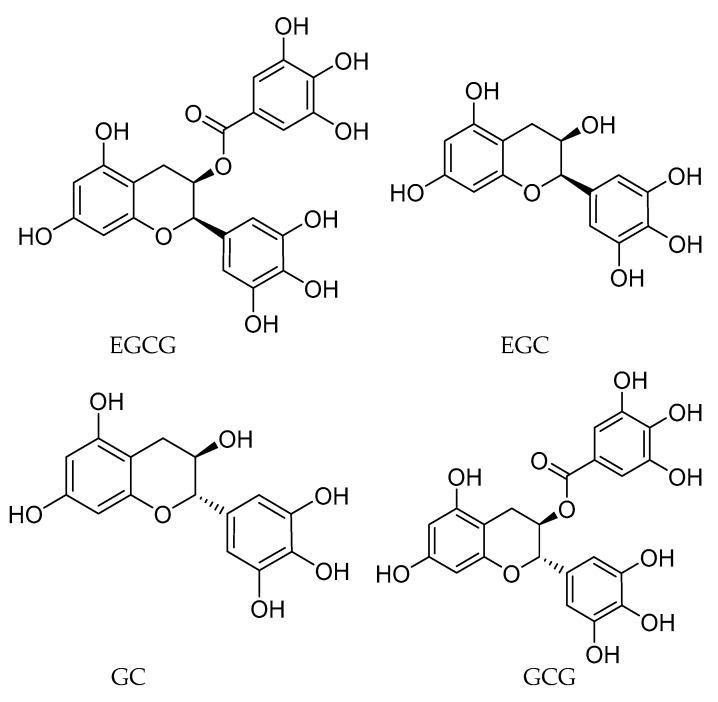
Structures of the green tea catechins assayed.

There is a strong evidence that topical treatments for CL have a high cure rates, safety and effectiveness, with low side-effects, relapse and recurrence rates [13]. A previous study has shown that EGCG formulated with USP hydrophilic ointment has a good penetration into mouse and human skin [14]. Therefore, taking into account all the mentioned above, the aims of this study were to assess in vitro the antileishmanial activity of green tea catechins (GTC) (Figure 1) against intracellular amastigotes, and to evaluate the efficacy of a topical treatment with EGCG, the most abundant flavanol in green tea, in a *L. (L.) amazonensis*-infected BALB/c mice model of CL.

## 2. Results

### 2.1. Antileishmanial Activity Against L. (L.) Amazonensis 

We investigated the activity of four GTC against intracellular amastigotes. These compounds showed antileishmanial activity in macrophages infected with *L. (L.) amazonensis* after 48 h of incubation, EGCG and EGC being the most active with IC_50_ values <59.6 µg/mL (Table 1). On the other hand, the IC_50_ of Glucantime (Gl), the reference drug for the treatment of leishmaniasis, was 6.5 µg/mL (Table 1). 

### 2.2. Citotoxicity

The cytotoxicity of GTC was also evaluated in vitro on THP1 cells (human leukemia monocytic cells line). Selectivity indexes showed that EGCG, GCG, EGC and GC were more selective against *Leishmania* amastigotes than the macrophage cells (SI > 1) (Table 1). Gl showed lower toxicity on THP1 cells than catechins, with a 50% cytotoxic concentration (CC_50_) >400 µg Sb^v^/mL. 

The selectivity index (SI) (CC_50_ for THP1/IC_50_ for *Leishmania*) was calculated. A SI higher than one, was considered more selective for activity against *Leishmania*, whereas a value lower than one was considered more selective for the activity against the host cells [15]. 

### 2.3. In Vivo Antileishmanial Activity of Catechin 

**Figure 2:**
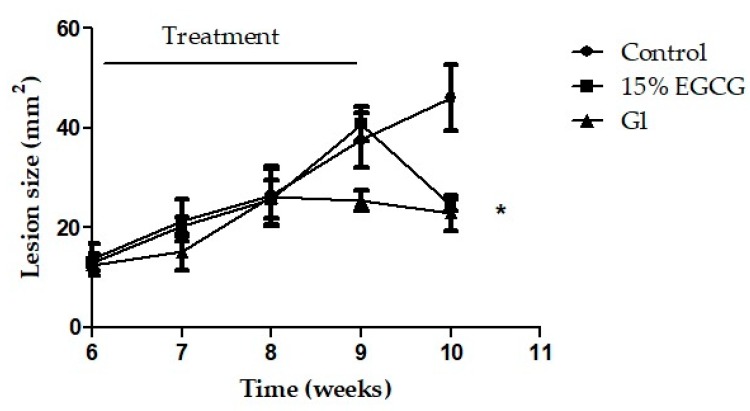
Evaluation of efficacy of topical treatment with 15% EGCG in BALB/c mice infected with *L. (L.) amazonensis*. Animals were treated with 15% EGCG during 18 days, once a day, six times a week and Gl was administered by intraperitoneal route, once a day, six times a week (120 mg Sb^v^/kg/d). Lesions’ sizes of mice treated with topical 15% EGCG decreased significantly at week 10 compared to control group (untreated mice) (*p* < 0.05). In addition, no difference in lesion size was observed between mice treated with 15% EGCG and Gl, the first-line drug for the treatment of leishmaniasis. Lesions’ sizes in the control group increased as was expected.

**Figure 3:**
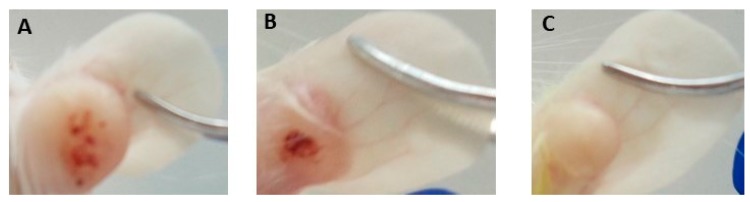
Promastigotes of *L. (L.) amazonensis* (5 × 10^3^/20 µL) were inoculated in the ear dermis of BALB/c mice. After six weeks postinfection, mice were treated with topical 15% EGCG and Gl. The photomicrographs are showing the efficacy of 15% EGCG and Gl at the end of the experiment (week 10). (**A**) Lesion with elevated border and central crater of an untreated mouse. (**B**) Small lesion with thin border of a mouse treated with 15% EGCG. (**C**) Small papular lesion after treatment with Gl.

**Figure 4:**
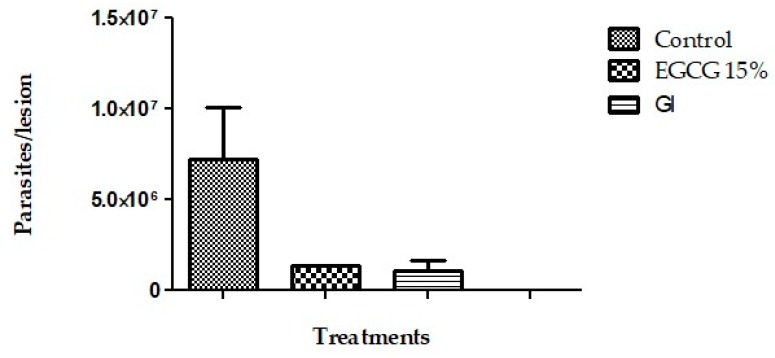
Efficacy of topical treatment with 15% EGCG in BALB/c mice infected with *L. (L.) amazonensis*. Parasite burden decreased significantly after 18 days of treatment with topical EGCG with respect to control group (*p* < 0.05). In addition, EGCG showed a similar efficacy to the intraperitoneal Gl treatment. Results represent the mean ± standard deviation of two independent experiments.

The activity of EGCG, the main GTC, was evaluated in an *L. (L.) amazonensis*-BALB/c model. No significant differences were observed in the lesions’ sizes when the treatments were initiated (Figure 2). After one week of topical treatment with 15% EGCG, the edge of the lesions began to flatten. At the end of the experiments (week 10), EGCG promoted a significant reduction of the lesion size (p < 0.05), showing smaller lesions than control group. In the same manner, treatment with Gl, the reference drug for leishmaniasis, reduced the lesion size as was expected (*p* < 0.05) (Figure 2). The changes observed in the evolution of lesions in mice treated with topical EGCG were correlated with a significant decrease of the parasite burden (80.4% of inhibition) compared with control group (p < 0.05) (Figure 3 and Figure 4). In addition, EGCG showed a similar efficacy to intraperitoneal treatment with Gl (85.1% of inhibition) (Figure 4). 

## 3. Discussion

The green tea from the leaf of *Camellia sinensis* is one of the most popular beverages worldwide, and for many years the health benefits of its PE fraction have been studied. The PE is constituted by catechins, and its biological activities, such us antimicrobial, anticancer and antiprotozoal, have been reported [3,4,5,6,7]. 

In a previous work, we showed the leishmanicidal activity of EGCG, EGC, GCG, GC and PE against promastigotes of *L. (L.) amazonensis* and *L. (V.) braziliensis*, being the former one, the more active catechin [6]. On the other hand, (+)-catechin, (−)-epicatechin and (−)-catechin gallate did not inhibit the growth of parasites in both species [8]. This prompted us to evaluate and compare the activity of EGCG, EGC, GCG and GC in macrophages infected with *L. (L.) amazonensis.* Among the catechins studied, ECGC and EGC were the most active ones against intracellular amastigotes with IC_50_ values <59.6 µg/mL. Other studies in vitro, reported the EGCG activity against intracellular amastigotes of *L. (L.) amazonensis* and *L. (V.) braziliensis* with IC_50_ values of 0.74 µg/mL and 1.57 µg/mL, respectively [10,11]. The low values of IC_50_ of EGCG, may be due to the experimental conditions used in the assays, such as the incubation temperature of infected macrophage cultures (37 °C). It is well known that amastigotes of *L. (L.) amazonensis* and *L. (V.) braziliensis* are sensitive to temperature [16]. Therefore, incubation at 37 °C can significantly inhibit parasite growth compared to that at 34 °C in the absence of antileishmanial drugs, giving a significantly lower IC_50_ in the presence of drugs [16]. On the other hand, EGCG and GCG showed antileishmanial activity against another species, *L. (L.) donovani,* the causal agent of visceral leishmaniasis, with IC_50_ values of 19.1 µg/mL and 8.9 µg/mL, respectively [17]. 

Besides the in vitro experiments, herein we performed an in vivo evaluation of the topical EGCG in hydrophilic ointment on *L. (L.) amazonensis*-BALB/c mice, since a topical treatment for CL is desirable due to the limited side effects and easy administration [13]. According to our knowledge, no previous study has reported the topical use of EGCG against CL. In addition, it was demonstrated that EGCG in hydrophilic ointment was efficiently absorbed into intact mouse skin with an uptake of up to 1%–20% of the applied dose [14]. In the present study, after 18 days of daily topical application with EGCG, both the lesions’ sizes and parasite burden were reduced significantly (80.4% inhibition) compared to the control group. Moreover, topical EGCG showed a similar efficacy to Gl, the reference drug for leishmaniasis treatment. It is important to mention that, in this study, the treatment with EGCG was initiated when the infection has already been established (week six post-infection). On the other hand, Inacio et al. reported the efficacy of oral treatment with EGCG in *L. (L.) amazonensis* in vivo model at the onset of the infection (week one post-infection); the treatment reduced significantly the parasite burden and lesion size compared with control group [10]. In addition, EGCG was also effective in mice infected with *L. (V.) braziliensis* but with a dose 3.3-fold higher (100 mg/kg/day) than the dose required for *L. (L.) amazonensis* (30 mg/kg/day) [10,11].

The antileishmanial activity of EGCG observed against different species of *Leishmania* in both the in vitro and in vivo model of infection can be explained by the inhibitory effect of EGCG on the enzyme arginase, involved in the polyamine synthesis pathway as demonstrated by dos Reis et al. [12]. Besides, this enzyme was considered a target to control *Leishmania* infection [12]. 

Thus, in the present work we demonstrated in vitro the antileishmanial activity of EGCG and EGC. Furthermore, topical treatment with EGCG showed a similar efficacy to Gl, the standard drug for treating leishmaniasis, in an animal model of cutaneous leishmaniasis caused by *L. (L.) amazonensis.*

## 4. Materials and Methods 

### 4.1. Compounds

(−)-Epigallocatechin gallate (EGCG), (−)-epigallocatechin (EGC), (−)-gallocatechin (GC) and (−)-gallocatechin gallate (GCG) were kindly supplied by Mitsui Norin (Shizuoka, Japan) and the purity of each compound was 98%. Meglumine antimoniate (Glucantime^®^, Aventis-Sanofi, São Paulo, Brazil) was kindly supplied by Ministerio de Salud de la Nación (Buenos Aires, Argentina).

### 4.2. Parasites

After several passages in mice, *L. (L.) amazonensis* (MHOM/BR/73/M2269) was isolated with sterile proline balanced salts solution (PBSS) containing 100 U/mL penicillin and 50 µg/mL streptomycin (P-E) and cultured in a Difco blood agar (USMARU) medium containing 20% defibrinated rabbit blood [18]. Then, the promastigotes were cultured in RPMI 1640 medium supplemented with 10% (*v*/*v*) heat-inactivated fetal bovine serum (FBS) and P-E. The parasites were maintained by successive passages at 23 °C. 

### 4.3. Macrophage Cytotoxicity Screening

The cytotoxicity of EGCG, EGC, GCG and GC was evaluated in a human monocytic cell line derived from an acute monocytic leukemia patient (THP1). The cells (3 x 10^4^/150 µL) were first differentiated to macrophages with 10 ng/mL of phorbol myristate acetate for 24 h at 37 °C in a 5% CO_2_ 95% air mixture. Then, the cells were treated with a different concentration (1.5 to 100 µg/mL) of EGCG, EGC, GCG and GC and Gl (12.5 to 400 µg Sb^v^/mL) during 48 h. The controls were incubated with RPMI 1640 medium (Gibco, Grand Island, NY, USA). The viability of the macrophages was determined by the MTT assay (Nacalai tesque, Kyoto, Japan). Briefly, 50 μL of MTT (2 mg/mL) was added to each well and, after 4 h of incubation at 37 °C, the reaction was stopped dissolving the formazan crystals with 100 μL/well of dimethyl sulfoxide (DMSO). The relative amount of formazan produced by viable macrophages was determined using a spectrophotometer at 570 nm. The percentage of viable cells was calculated according to the formula: OD treated culture–OD blank x 100/OD control–OD blank as was described previously [19]. The CC_50_ was calculated for each compound as the concentration of compound that reduces cell growth by 50%.

### 4.4. In Vitro Antiamastigote Activity

THP1 cells (2 × 10^5^/mL) were differentiated to macrophages with 10 ng/mL of phorbol myristate acetate and placed in Lab-Tek eight chamber slides for 24 h at 37 °C in a 5% CO_2_ 95% air mixture. Adherent macrophages were infected with *L. (L.) amazonensis* at a ratio of four parasites per macrophage and incubated at 34 °C in a 5% CO_2_ 95% air mixture during 24 h [16]. After infection, the cells were washed with pre-warmed PBS to remove free parasites. A new medium with different concentrations of EGCG, EGC, GCG and GC (2.3 to 130 µg/mL) and Gl (2.5 to 80 µg Sb^v^/mL) was added to each well. The positive controls were infected cells cultured under the presence of complete RPMI-1640 medium. The chambers were returned to the CO_2_ incubator for an additional 48 h at 34 °C, 5% CO_2_ [17]. The cells were stained with Diff Quik (Biopur S.R.L., Rosario, Argentina), and the results were expressed as the concentration of compound to inhibit the 50% of the growth (IC_50_) of the intracellular amastigotes.

### 4.5. Animals

BALB/c mice were bred at the mouse facility of Instituto de Patología Experimental, Faculty of Health Sciences, National University of Salta (Salta, Argentina). All procedures adopted in this study were according to ethical standards and the protocol was approved by the Animal Ethics Committee of the Faculty of Health Sciences, National University of Salta (Salta, Argentina) (Resolution 309–18). 

### 4.6. In Vivo Studies

Female BALB/c mice of six weeks old (weight, 20 to 25 g) were randomly separated into three groups of five animals and were inoculated by intradermal via in their right ear with 5 × 10^3^ (20 µL) infective promastigotes of *L. (L.) amazonensis* (late log phase of growth). The animals were housed in groups of five in metal cages under a controlled environmental condition (12:12 h light/dark cycle and 23 °C room temperature) with food and water ad libitum.

At week six postinfection, mice received the treatments as follow: group 1, treated with EGCG in USP hydrophilic ointment (15%), group 2, treated with 120 mg Sb^v^/kg/d of Gl administered by intraperitoneal route, and group 3, control mice (without treatment). EGCG was uniformly mixed in USP hydrophilic cream (Yoshida Pharmaceutical Co., Saitama, Japan) (15% w/w) and was topically applied (20 mg/lesion). EGCG ointment was prepared daily. The treatments were given during 18 days, once a day, six times a week, until the end of the treatments (9^th^ week). The efficacy of treatments was determined by measuring the lesions’ sizes (mm^2^) with a digital caliper weekly (150 mm; Digimess) and by quantifying the parasite burden with the limit dilution assay as was described previously [20]. 

### 4.7. Statistical Analysis

The GraphPad Prism 5.0 software (GraphPad Software Inc., San Diego, CA, USA) was employed to carry out calculations of the IC_50_ and CC_50_ values by a nonlinear regression. Multiple comparisons of lesions’ sizes between groups were made with a one-way analysis of variance (ANOVA) followed by Tukey test. The statistical analysis of the parasite burden data obtained by the limiting dilution assay was done using ELIDA software, which analyses data through the Poisson distribution and by the X^2^ test [21]. *p* < 0.05 was considered significant. The data are representative of two separate experiments in vivo.

## 5. Conclusions 

The present work demonstrated the antileishmanial activity of EGCG and EGC were the most active catechins against intracellular amastigotes in an in vitro model. Moreover, topical EGCG showed a similar efficacy to the Gl treatment, promoting a significant size reduction in lesions and the parasite burden in an animal model of cutaneous leishmaniasis caused by *L. (L.) amazonensis*. In order to improve even more the efficacy of a topical treatment with EGCG, the synergism between EGCG and ECG should be evaluated in further studies.

## Figures and Tables

**Table 1:** Antileishmanial activity against intracellular amastigotes of *L. (L.) amazonensis,* cytotoxicity and selectivity index of green tea catechins after 48 h of treatment.

Compounds	Intracellular Amastigotes IC_50_ (µg/mL)	THP1 CC_50_ (µg/mL)	SI
**EGCG**	59.6 ± 9.3	88.9 ± 21.9	1.5
**EGC**	≥44.3	120.7 ± 20	≥2.7
**GCG**	67.5 ± 9.5	94.9 ± 11.8	1.4
**GC**	93.5 ± 5.0	115.2 ± 37.1	1.2
**Gl**	6.5 ± 1.7	>400	>61.5

IC_50_: inhibitory concentration for 50% of parasites; CC_50_: cytotoxic concentration for 50% of cells; SI: selectivity index obtained from ratio CC_50_ THP1/IC_50_ *Leishmania*. Results represent the mean ± standard deviation of two independent experiments by duplicate.
